# Cortical Source Multivariate EEG Synchronization Analysis on Amnestic Mild Cognitive Impairment in Type 2 Diabetes

**DOI:** 10.1155/2014/523216

**Published:** 2014-08-28

**Authors:** Dong Cui, Jing Liu, Zhijie Bian, Qiuli Li, Lei Wang, Xiaoli Li

**Affiliations:** ^1^School of Information Science and Engineering, Yanshan University, Qinhuangdao 066004, China; ^2^School of Electrical Engineering, Yanshan University, Qinhuangdao 066004, China; ^3^Department of Neurology, General Hospital of Second Artillery Corps of PLA, Beijing 100875, China; ^4^State Key Laboratory of Cognitive Neuroscience and Learning and IDG/McGovern Institute for Brain Research, Beijing Normal University, Beijing 100875, China; ^5^Center for Collaboration and Innovation in Brain and Learning Sciences, Beijing Normal University, Beijing 100875, China; ^6^National Key Laboratory of Cognitive Neuroscience and Learning, Beijing Normal University, Beijing 100875, China

## Abstract

Is synchronization altered in amnestic mild cognitive impairment (aMCI) and normal cognitive functions subjects in type 2 diabetes mellitus (T2DM)? Resting eye-closed EEG data were recorded in 8 aMCI subjects and 11 age-matched controls in T2DM. Three multivariate synchronization algorithms (*S*-estimator (*S*), synchronization index (SI), and global synchronization index (GSI)) were used to measure the synchronization in five ROIs of sLORETA sources for seven bands. Results showed that aMCI group had lower synchronization values than control groups in parietal delta and beta2 bands, temporal delta and beta2 bands, and occipital theta and beta2 bands significantly. Temporal (*r* = 0.629; *P* = 0.004) and occipital (*r* = 0.648; *P* = 0.003) theta *S* values were significantly positive correlated with Boston Name Testing. In sum, each of methods reflected that the cortical source synchronization was significantly different between aMCI and control group, and these difference correlated with cognitive functions.

## 1. Introduction

The incidence and prevalence of diabetes mellitus (DM) are increasing worldwide, especially with an increase in type 2 diabetes mellitus (T2DM), which represents more than 90% of all cases of diabetes [1]. T2DM has become a significant public health problem [2, 3]. Many recent studies indicated that T2DM was a risk factor for cognitive impairment [4, 5] and may cause alteration of EEG characteristics related to cognitive functions [6, 7]. Mild cognitive impairment (MCI) is characterized as an early but measurable stage of cognitive impairment [8, 9]. MCI is predictive of progression to dementia and the conversion rate from MCI to dementia was 11.65% per year [10]. One study suggests that individuals with diabetes are 1.5 times more likely to experience cognitive decline and have 1.6 times greater risk of future dementia than individuals without diabetes [11].

A promising approach to study MCI subjects is to analyze the recordings of electroencephalographic (EEG). EEG has been applied to discriminate healthy subjects with various types and severities of cognitive impairment for many years [12]. This technique is based on low cost and relatively widely available equipment in the majority of countries and is fully noninvasive. Considering the fact that MCI responds much better when treatment is commenced in the early stage of the illness than in the advanced stages, investigating the EEG signal characteristics related to MCI of diabetes patients is very important for us to understand the specific effect of brain structure and functional connective in diabetes [13].

At present, some studies analyzed the EEG recordings to study the pathophysiology of MCI in DM. Cooray et al. studied event-related potentials (ERPs) and resting EEG power spectrum in the patients with T2DM and healthy control subjects. The T2DM affected ERP with a decrease in N100 amplitude and an increase in P300 latency. T2DM patients had an increase in alpha-power and increase in connectivity in the alpha and theta bands across hemispheres and within lateral regions. Moreover, T2DM patients had a significant increase in connectivity in the beta band of the central region [1]. Hazari et al. studied P300 ERPs in T2DM patients without clinical evidence of central nervous system damage and nondiabetic controls. They found that P300 ERPs revealed cognitive dysfunction, which was not detected by neuropsychometric test and if the disease duration exceeds 5 years the decrease became more prominent [3].

Some studies analyzed the EEG synchronization of MCI and Alzheimer's disease (AD) but not diabetes patients. The AD patients showed lower synchronization values in beta band compared with MCI patients [14]. At group level, frontoparietal coupling of the delta and alpha rhythms progressively became abnormal through MCI and mild AD [15]. Compared with the age-matched healthy controls, AD patients had significant lower synchronization values in the beta1, beta2, and beta3 bands, and they were significantly correlated to scores of symptom severity scales [16].

An advanced method for analyzing neural activity sources (low resolution brain electromagnetic tomography (LORETA)) had been successfully applied to studying on normal and pathological aging [17]. Babiloni et al. firstly investigated the power spectrum profiles at the level of cortical EEG sources with LORETA in mild AD patients, vascular dementia (VaD), and normal elderly people (Nold) groups and they found that alpha sources (8–10 Hz) were abnormal in mild AD compared with Nold and VaD subjects [18]. Then, Babiloni et al. found that the frontal white matter and the amplitude of frontal delta sources (2–4 Hz) were negatively correlated across MCI and AD subjects [19]; the alpha sources in parietal, occipital, and temporal areas were lower in amplitude in the AD and MCI subjects than in the Nold subjects, while the magnitude of the delta EEG sources was higher in the AD than in the Nold and MCI subjects [20, 21]. In addition, standardized low resolution electromagnetic tomography analysis (sLORETA) allowed 3D localization of standardized current density with zero localization errors in both the time and frequency domain [22]. Caso et al. indicated that AD patients had higher theta power with scalp EEG spectral and lower alpha2 and beta1 values in central/temporal regions with cortical sources EEG rhythms by sLORETA than frontotemporal dementia (FTD) subjects [23]. Compared with the normal elderly subjects, the cognitive impairment subjects showed a decrease in amplitude of the alpha1 sources and an increase in amplitude of the delta sources [24].

However, little is known about the synchronization activity of EEG sources with amnestic MCI (aMCI) in T2DM. As far as we know, this paper is the first study about cortical source multivariate EEG synchronization analysis on aMCI in type 2 diabetes.

In the study, we recorded the resting EEG data of aMCI and nonamnesic MCI patients in T2DM. The EEG sources in time domain were estimated by sLORETA. The synchronization values of cortical EEG sources were calculated by using four kinds of multivariate synchronization methods for each subject. Then statistics analysis was done to explore the difference of the synchronization between aMCI group and control group (nonamnesic MCI patients). We also analyzed the correlation between synchronization and cognitive functions.

## 2. Methods

### 2.1. Subjects and Diagnostic Criteria

Nineteen T2DM patients were recruited from the Neurology Department of Second Artillery General Hospital of Beijing in China. Diagnosis and classification criteria were recommended by the World Health Organization [25]. The study was approved by Beijing Normal University ethics committee. Written informed consent was obtained from all participants before the experiment. The experiment was conducted in accordance with the Declaration of Helsinki (1964). All the patients had sufficient visual and auditory resolution to accept the neuropsychological test.

The aMCI group consisted of 8 subjects and control group consisted of 11 subjects. Inclusion criteria of the aMCI group were (1) the reporting of a decline in cognitive functioning relative to previous abilities during the past year by the patient or their families, (2) cognitive disorders as evidence by clinical assessment (with the hypomnesia of chief complaint, or in another cognitive domain, which in this study was assessed by neuropsychological test such as MMSE and MoCA, and (3) the activity of daily living being unaffected by history and evidence of independent living as assessed by Instrumental Activity of Daily Living Scale test [26].

Exclusion criteria for aMCI group were (1) MCI subjects without objective memory deficits; (2) having any other psychiatric or neurological disorders such as AD, dementia, Parkinsonism, depression, extrapyramidal disease, brain trauma, brain tumor, epilepsy, and neuropathic recession; (3) suffering from severe physical illness, such as the clinical macrovascular complications (angina, history of myocardial infarction, cerebral infarction with clinical symptoms and peripheral vascular embolism, etc.). (4) patients with severe alcoholism or drug abuse; (5) taking the sedative-hypnotic drugs and some drugs that affect the central nervous system one month before joining the group.

The control group inclusive criteria were (1) no memory impairment chief complaint; (2) activities of daily living without defect; (3) normal cognitive functions as evidenced, assessed by neuropsychological test such as MMSE and MoCA. Exclusion criteria for control group were the same as (2)–(5) items of the exclusion criteria for aMCI group.

The data including patients' name, age, gender, education years, and handedness were recorded. A series of standardized neuropsychological tests were used to assess the cognitive functions of aMCI and control groups. Each patient was evaluated by the Mini-Mental State Examination (MMSE) and Montreal Cognitive Assessment (MoCA). Attention and executive function were examined by the Wechsler Adult Intelligence Scale (WAIS) Digit Span Test [27] and the Trail Making Test parts A and B. Tests to assess memory were immediate and delayed recall measure and the delay recognition measure of the Rey Auditory Verbal Learning Test (AVLT) [28]. For language, semantic fluency test of the one minute verbal fluency for animals and Boston Naming Test [29] were used. The activity of daily living was tested by Instrumental Activity of Daily Living Scale (IADL) [30].

The demographic characteristics and neuropsychology of the aMCI group and the control group were investigated and statistically analyzed by independent samples *t*-test analysis with SPSS20.0. The results were shown in Table 1. The demographic data had no significant difference between the two groups. All subjects were matched regarding age, gender, and education level. Neuropsychological tests showed that the scores of MMSE and MoCA achieved a significant level (*P* value < 0.05). The memory functions and language fluency were better preserved in control group than in aMCI group. The executive function and attention and daily living abilities were not significantly different between the two groups, except for the WAIS digit span test.

### 2.2. EEG Recording and Preprocessing

The patients were instructed to sit relaxed in a dim quiet room of Department of Neurology, General Hospital of Second Artillery Corps of PLA, Beijing, China. The room temperature was kept at 23 ± 2°C. The resting-state eyes-closed EEG signals were recorded using EGI system of Net Amps 300 amplifiers (Electrical Geodesics Inc. (EGI), Eugene, OR). High-density 128-channel EEG data were recorded for 5 minutes using the vertex sensor (Cz) as the reference electrode. All electrode impedances were kept under 50 kΩ. The signals were digitized at a rate of 1000 samples per second with a 0.10 Hz~200 Hz band-pass filter. Artifacts in all channels, such as electrooculogram (EOG) and electromyogram (EMG), were eliminated visually by expert electroencephalographers (blind to the diagnosis).

### 2.3. Cortical Sources Analysis of the EEG Rhythms by sLORETA

Standardized low resolution electromagnetic tomographic analysis (sLORETA) is a functional imaging technique belonging to a family of standardized linear inverse solution procedures. sLORETA can model 3D distributions of the cortical sources based on extracranial measurements (i.e., scalp EEG) that would provide important information on the time course and localization of brain function [22]. sLORETA solutions are computed within a three-shell spherical head model including scalp, skull, and brain compartments. The brain compartment is restricted to the cortical gray matter/hippocampus of a head model coregistered to the Talairach probability brain atlas, which has been digitized at the Brain Imaging Center of the Montreal Neurological Institute [31]. The brain compartment includes 6239 voxels with 5 mm resolution. The electrode coordinates were the average location of the 10-5 system [32]. sLORETA has been successfully used in EEG and MEG analysis [33, 34].

In order to achieve greater confidence in the sLORETA solutions and synchronization estimates in the study, the artifact-free normalized EEG data were segmented into nonoverlapping 5-second epochs. Then the epochs of the aMCI group and control group were given as an input to the original sLORETA software (http://www.uzh.ch/keyinst/loreta.htm). The amplitudes of current density as time series on 6239 voxels were calculated. For each Brodmann area, only a single voxel, which was closest to the center of the Brodmann area, was selected for the reasons that the spatial resolution of sLORETA was low; thus the single centroid voxel is an excellent representative of each Brodmann area. In addition, if the average sLORETA solutions were used, the sLORETA activity spanning a large volume might not be very meaningful. In the study, five ROIs (frontal, central, parietal, temporal, and occipital) were achieved. Each of the ROIs was made of corresponding Brodmann areas [35] (Table 2). Thus we had 58 channels' data according to these Brodmann areas.

### 2.4. Synchrony Measures

In this paper, we calculated the synchronization values of each 5 s sLORETA cortical sources epoch by using four multivariate synchronization methods. Three methods were used to measure synchronization of the five ROIs in the following frequency bands: delta (1.5–6 Hz), theta (6.5–8 Hz), alpha1 (8.5–10 Hz), alpha2 (10.5–12 Hz), beta1 (12.5–18 Hz), beta2 (18.5–21 Hz), beta3 (21.5–30 Hz), gamma1 (30–45 Hz), gamma2 (55–80 Hz), and full (1.5–80 Hz) for all subjects.

#### 2.4.1. *S*-Estimator

*S*-estimator is a kind of global synchronization index based on principal component analysis (PCA) [36]. It can measure the synchronization by measuring the eigenvalue in the correlation matrix of a multivariate set of signals and has been applied to analysis synchronous about multichannel EEG signals. The *S*-estimator is defined as

(1) $$\begin{array}{c} S=1+\frac{\sum_{i=1}^{M}\lambda_{i}^{\prime }\log\left(\lambda_{i}^{\prime }\right)}{\log\left(M\right)}, \end{array}$$

where *λ*_*i*_ is the eigenvalue obtained from the equal-time correlation matrix **C** = [*c*_*ij*_] [37]. In the study, *c*_*ij*_ = |〈exp⁡⁡(*i*(*φ*_*i*_(*t*)−*φ*_*j*_(*t*)))〉_*t*_|, *i*, *j* = 1,…, *M*, and *φ*_*i*_(*t*) is the instantaneous phase of channel *i* for a frequency band. *φ*_*i*_(*t*) is extracted by Gabor wavelet transform. The *λ*_*i*_′ = *λ*_*i*_/∑_*i*=1_^*M*^ *λ*_*i*_ are the normalized eigenvalues. *M* means the channel number in the original signals. When all normalized eigenvalues are roughly of the same value, *S* is close to zero. That is to say, the signals are statistically independent. On the contrary, if the signals are well synchronized, only a few number of eigenvalues remain prominent, and, as a result, *S*-estimator is then close to one.

#### 2.4.2. Synchronization Index (SI)

By comparing the eigenvalues of data correlation matrix **C** and surrogate correlation matrix  **R**, synchronization index was used to measure the synchronization of multiple neural mass populations [38]. The surrogate data were obtained with amplitude-adjusted Fourier transform (AAFT) [39] method, which randomized the phase of time series. The surrogate data not only keeps the linear properties of itself in time series but also eliminates the nonlinear correlation between multivariable time series.

The normalized SI is computed using the following equation:

(2) $$\begin{array}{c} \text{S}{\text{I}}_{k}=\{\begin{array}{cc} \frac{\left(\lambda_{k}-\overset{-}{\lambda_{k}^{s}}\right)}{\left(M-\overset{-}{\lambda_{k}^{s}}\right)} & \text{if}\;\;\lambda_{k}>\left(\overset{-}{\lambda_{k}^{s}}+P\times \text{S}{\text{D}}_{k}\right)\;\;k=1,\ldots ,M\\ 0 & \text{otherwise}, \end{array} \end{array}$$

where *M* represents the number of time series, and *λ*_*k*_ represent the eigenvalues obtained from matrix** C**, which is constructed as mentioned above. $\overset{-}{\lambda_{i}^{s}}$ and SD_*k*_ denote the mean and standard deviation values of the eigenvalues of matrix **R**. And *P* is a constant that determines the threshold. In the study, *P* = 3 was used for a confidence probability of 0.95. Here we focus on the largest SI and refer to it as the SI value, and SI value ranges from 0 to 1. If SI = 0, it means no synchronization over all the series, and high SI values can be interpreted as the presence of increased connectivity over all the series. If SI = 1, it means that all series are complete synchronization.

#### 2.4.3. Global Synchronization Index (GSI)

Based on the description of the SI method, a new method of measuring global synchronization of multiple time series has been proposed [38]. We used the equal-time correlation method to get the correlation matrix **C**, the surrogate correlation matrix **R**, and their eigenvalues, like the SI method. In the GSI method, the eigenvalues of matrix **C** are normalized by dividing the eigenvalues of matrix **R** to reduce the influence of random component. The GSI is defined as

(3) $$\begin{array}{c} \text{GSI}=1+\frac{\sum_{i=1}^{M}\lambda_{i}\log\left(\lambda_{i}\right)}{\log\left(M\right)}, \end{array}$$

where *λ*_*i*_ is the normalized eigenvalues and computed as

(4) $$\begin{array}{c} \lambda_{i}=\frac{\left(\lambda_{i}/\overset{-}{\lambda_{i}^{s}}\right)}{\sum_{i=1}^{M}\lambda_{i}/\overset{-}{\lambda_{i}^{s}}},\;i=1,\ldots ,M, \end{array}$$

where $\overset{-}{\lambda_{i}^{s}}$ represent the mean values of the eigenvalues of matrix **R**. *M* represents the number of time series like the SI mentioned. To understand how this estimator works, two cases should be considered. If there is no genuine correlation, the normalized eigenvalues are all equal to 1/*M*, so GSI = 0; on the contrary, if all the time series are correlated completely, the largest normalized eigenvalue should be *M*, and the others are equal to zero; at this point, GSI = 1.

### 2.5. Statistics Analysis

Independent samples *t*-test was performed in demographic tests, MMSE scores, MoCA scores, and neuropsychological tests between aMCI and control group. In the paper, the statistical analysis was used by SPSS 20.0 software (IBM SPSS, Inc., NY, Armonk, USA).

Repeated measurement of analysis of variance (ANOVA) was performed on regional synchronization values: within-subject factors were “band” (delta, theta, alpha1, alpha2, beta1, beta2, beta3, gamma1, gamma2, and whole frequency band) and “ROIs” (frontal, central, temporal, parietal, and occipital), while between-subject factor was “group” (aMCI and control group). Age, education level, and gender were used as covariates in the statistical analysis to reduce confounding effects. Mauchly's test was used to evaluate sphericity of data; correction of the degrees of freedom was made by Greenhouse-Geisser procedure. Post hoc least significant difference (LSD) tests were used to compare group differences for each rhythm and ROIs.

Pearson's linear correlations were used between regional synchronization values and neuropsychological tests for all the subjects (aMCI and control group).

## 3. Results

### 3.1. Statistical Comparisons of the Synchronization Values

**Figure 1:**
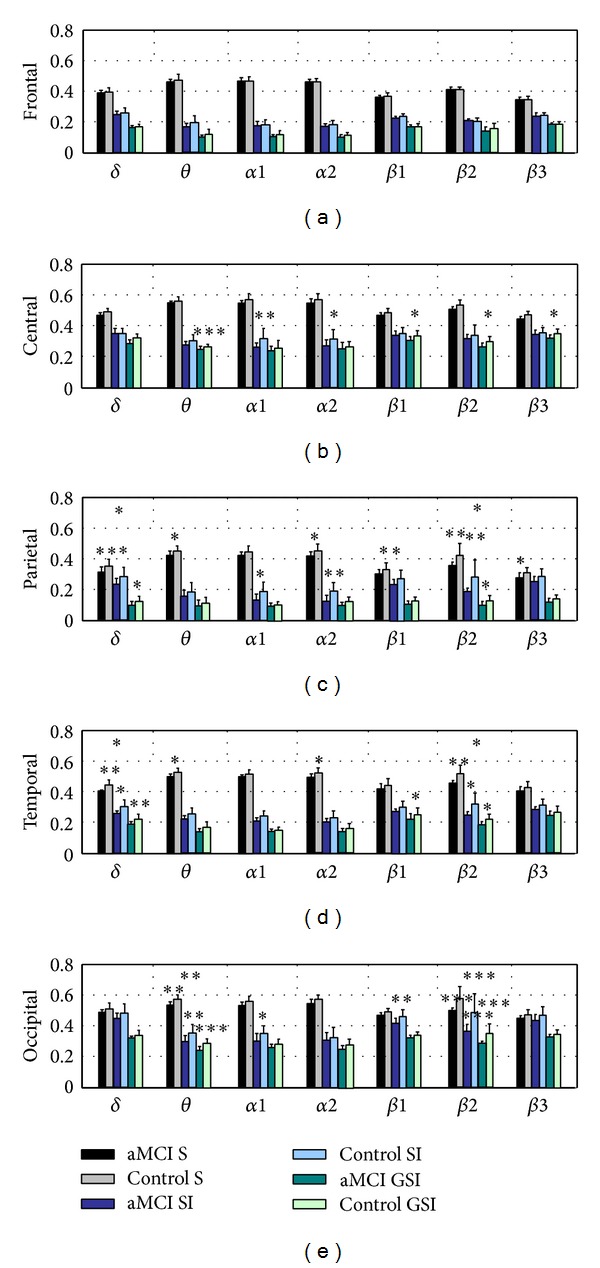
Regional synchronization values (mean ± standard error) of delta, theta, alpha1, alpha2, beta1, beta2, and beta3 bands which were calculated by the *S*-estimator, SI, and GSI method in aMCI and control group. Post hoc analysis results were graphically displayed inside the horizontal bar over the graph: asterisks were referred to aMCI/control comparison: one asterisk means *P* < 0.05, two mean *P* < 0.01, and three mean *P* < 0.001.

Figure 1 showed the bar graphs of regional synchronization values which were calculated by the *S*-estimator, SI and GSI, and the results of statistical analysis. The three synchronization measures of aMCI group decreased in all bands relative to a statistical interaction among the factors, which were the groups (aMCI and control group), bands (delta, theta, alpha1, alpha2, beta1, beta2, and beta3), and ROIs (frontal, central, temporal, parietal, and occipital). The figure did not show gamma1, gamma2, and the whole frequency band because synchronous values in these bands were not significantly different between two groups.

In Figure 1, frontal *S*, SI, and GSI values of aMCI group were not significantly different. The three synchronization values in parietal and temporal regions all were significantly different in delta and beta2 bands between aMCI and control group. The figure also showed that occipital theta and beta2 synchronization values measured by the three methods were more significantly different between the two groups, especially in the beta2 band.

Performing the repeated measurement of ANOVA on *S* values, the group effect (*F*(1,19) = 15.912, *P* < 0.001), interaction Band∗ROI effect (*F*(36,36) = 13.442, *P* < 0.001) and interaction Band∗Group effect (*F*(9,162) = 7.844, *P* < 0.001) were significant. The interaction Band∗ROI∗Group was not significant (*F*(36,648) = 1.253, *P* = 0.223). For SI values, the group effect (*F*(1,19) = 16.046, *P* < 0.001), interaction Band∗ROI effect (*F*(36,36) = 4.324, *P* < 0.001), interaction Band∗Group effect (*F*(9,162) = 7.630, *P* < 0.001) and interaction Band∗ROI∗Group effect (*F*(36,648) = 1.662, *P* = 0.035) were significant. And for the GSI values, the group effect (*F*(1,19) = 15.617, *P* < 0.001), interaction Band∗ROI effect (*F*(36,36) = 15.739, *P* < 0.001), interaction Band∗Group effect (*F*(9,162) = 5.091, *P* < 0.001) were significant. The interaction Band ∗ROI∗Group was not significant (*F*(36,648) = 1.085, *P* = 0.363).

### 3.2. Correlation Analysis

**Figure 2:**
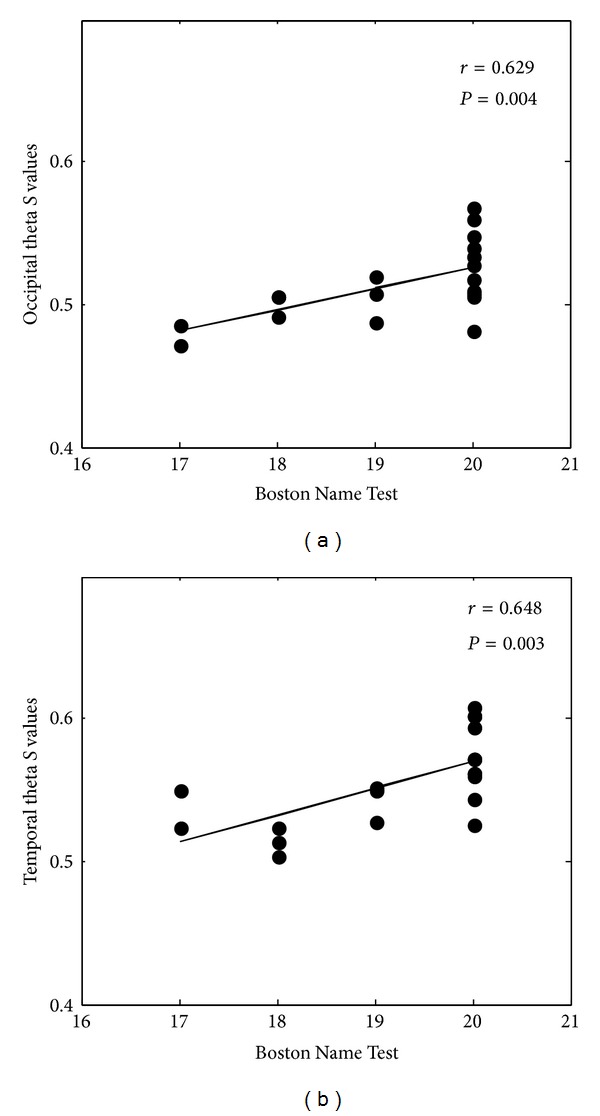
Correlation analysis between cortical source EEG synchronous and the scores of the neuropsychological tests in all subjects as a whole group (only significant correlations were displayed). *S* values as a function of Boston Name Test within temporal theta and occipital theta were reported; the correlation was *r* = 0.629, *P* = 0.004 and *r* = 0.648, *P* = 0.003, respectively.

In order to investigate the relationships between cognitive functions and the synchronization values that are computed by the *S*-estimator, SI, and GSI method respectively, Pearson's correlation analysis was performed between the synchronization values (different bands and ROIs) and the scores of neuropsychological tests (MoCA, AVLT immediate recall, AVLT delay recall, AVLT delay recognition, WAIS digit span test, semantic fluency, Boston Name Testing, Trail Making test A, and Trail Making test B) in all subjects as a whole group. Based on Bonferroni method, the significant correlation values *r* and *P* values (*P* < 0.01) were corrected. We found that only temporal theta *S* values (*r* = 0.629, *P* = 0.004), occipital theta *S* values (*r* = 0.648, *P* = 0.003), and Boston Name Testing were strict correlated significantly. Scatter grams were illustrated in Figure 2.

## 4. Discussion

In this paper, we studied the synchronizations of resting cortical sources with multichannel synchronization algorithms to analyze the synchronization mechanism of the amnestic MCI subjects in T2DM. To our knowledge, we firstly applied the source EEG synchronization with multichannel method in amnestic MCI with T2DM.

Our results showed that the synchronization values estimated by *S*-estimator, SI, and GSI methods decreased significantly in beta band and were similar to those of MCI and AD but not diabetes patients [40]. The synchronization values also decreased significantly in delta and theta bands of aMCI group in T2DM; it may be due to the source EEG synchronization or due to the fact that the patients were T2DM. We also found that in aMCI groups the parietal, temporal, and occipital networks show reduced synchronization observably in these three methods, while central networks reduced observably except in *S*-estimator. The synchronization values in frontal area reduced but all of them have no significance. Recent studies in MCI and AD but not diabetes patients showed that cortical sources of EEG rhythms were abnormal in temporal [23, 41], frontal, parietal, central, and occipital regions [35, 42], and the synchronization shows different in parietal, frontal [43, 44], temporal, and occipital regions [45].

In these three methods, each measure reflected different aspects of change in brain synchronization during amnestic mild cognitive impairment in T2DM. We also found that these methods have something in common; the results of three methods showed that aMCI group has lower synchronization values than control groups basically in all bands and ROIs, especially in parietal, temporal, and occipital regions. In these three methods, the occipital beta2 synchronization values were significantly lower in aMCI group than in control group.

At present, several studies have successfully analyzed the synchronization characteristics between multiple neural signals in MCI and AD patients but not diabetes patients. Pijnenburg et al. investigated the behavior of synchronization likelihood (SL) of multichannel EEG in AD, MCI, and cognitively healthy controls, both at rest and during a working memory task. Decrease of beta (12–30 Hz) band synchronization occurs in mild AD, both in a resting condition and during a working memory task [40]. Direction of information flux within EEG functional coupling at electrode pairs was performed by directed transfer function (DTF) in normal elderly, amnesic MCI, and mild AD subjects at rest condition (closed eyes). Parietal to frontal direction of the information flux within EEG functional coupling was stronger in normal elderly than in MCI and/or AD subjects, namely, alpha and beta rhythms. In contrast, the directional flow within interhemispheric EEG functional coupling did not discriminate among the three groups. These results suggest that directionality of parietal-to-frontal EEG synchronization is abnormal not only in AD but also in amnesic MCI [43]. Lee et al. analyzed the EEG of AD patients by using the global synchronization index, which is the same as SI method in this paper. They found that the GSI values in the beta1 (13–18 Hz), beta2 (19–21 Hz), beta3 (22–30 Hz), and gamma (30–50 Hz) bands were significantly lower in AD patients than in normal control. GSI values in the beta and gamma bands were positively correlated with the MMSE scores in all participants (AD and normal control) [16]. These studies indicated abnormal strength and direction of synchronization in MCI and AD patients.

The results of cortical sources analysis showed that the delta cortical sources in the whole brain, alpha cortical sources in occipital areas [46], and theta cortical sources in parietal and occipital were inordinate obviously in MCI and AD but not diabetes patients [35, 41]. However, surface topography provides limited information about source brain structures causing the changes in EEG synchronization. The synchronizations of cortical sources may be more accurate than the synchronizations of scalp EEG. Knyazeva et al. used local autoregressive average (LAURA) method to generate the sources of scalp EEG in AD patients and age-matched controls one-year follow-up. The main effect of AD was hyposynchronization in the medial temporal and frontal regions and relative hypersynchronization in posterior cingulate, precuneus, cuneus, and parietotemporal cortices. Rapidly progressing AD patients showed a significant reduction in synchronization with time, manifest in left frontotemporal cortex. Thus, the evolution of source EEG synchronization over time is correlated with the rate of disease progression and should be considered as a cost-effective AD biomarker [44].

We tried to figure out some statistically significant correlation between source of EEG synchronization and cognitive functions of patients revealed by neuropsychological test. The *S*, SI, and GSI values of theta or beta correlated positively with the Boston Name Testing, and the *S*, SI, and GSI values of delta or beta correlated positively with WAIS digit span test. Delta and beta GSI values and alpha SI values positively correlated with MoCA scores. Based on Bonferroni method, only temporal theta *S* values (*r* = 0.629, *P* = 0.004), occipital theta *S* values (*r* = 0.648, *P* = 0.003), and Boston Name Testing were strict correlated significantly.

Several studies used coherence as a measure of interdependencies between EEG or MEG data and found a significant loss of alpha band coherence in AD [47, 48]. Babiloni et al. pointed out that the intensity of alpha value changes in pathological aging as a function of the global cognitive level [49]. The beta band has classically been related to excitatory activity and cognitive processes that deteriorate during AD [14]. Furthermore, beta spectral power has also been shown to decrease in AD and the decrease of beta band synchronization is related to diminished semantic memory function [50]. Koenig et al. found that the delta band synchronization increased almost continuously with increasing cognitive impairments [14], while Jelic et al. reported that activity in AD patients reduces in delta and theta [51]. However, there are still controversial findings concerning coherence in the delta and theta bands, with some studies finding a decreased coherence and others finding an increased coherence [52].

The mechanisms of cognitive impairment in diabetes remain to be elucidated. Chronic hyperglycemia was one of the determinants of cognitive decline in people with T2DM [53]. Hyperglycemia may be directly toxic to the neuron, leading to its degeneration [54]. The deleterious effects of hyperglycemia were mediated through an increased influx of glucose through the polyol pathway forming sorbitol and fructose, oxidative stress, and nonenzymatic glycation of biomolecules resulting in advanced glycation end products (AGE) [53]. Hypoglycemia and hemodynamic changes in persons with T2DM are also associated with cognitive impairment, including lacunae and microinfarcts [55]. A few studies showed that patients with T2DM have increased arterial stiffness [56] associated with cognitive impairment [57]. Long duration of DM may increase the risk of cognitive dysfunction, causing atherosclerosis, stroke, and cerebral infarctions [58]. Studies have suggested that duration of diabetes, use of glucose-lowering medications, and degree of glucose control may modulate the risk of cognitive impairment in older persons with T2DM [59]. Insulin resistance contributes through the indirect mechanism of upregulating hypothalamic-pituitary-adrenal axis, thereby causing hypercortisolemia related cognitive dysfunction [60]. Hypertension usually exists as a comorbid condition with DM and may be a part of a larger metabolic syndrome, including hyperglycemia, hyperinsulinemia, and dyslipidemia. Hypertension and diabetes, when combined, increase the risk of cognitive impairment [61]. Other likely mechanisms of cognitive dysfunction in T2DM were extensive leukoaraiosis (white matter hyperintense lesions, WMHLs), atrophy in the region of hippocampus, and amygdala [62]. Reduced volumes of the amygdala and the hippocampus in T2DM patients may underlie deficits associated with learning and memory [55]. A report on MRI abnormalities and cognitive changes found substantial white matter lesions and subcortical atrophies in T2DM patients.

Our study had several limitations. First, our sample was relatively small, only 19 subjects in T2DM. Making additional studies with larger samples is necessary to generalize our conclusions. Second, there were no normal elderly subjects or AD patients included. The comparison of the synchronization values was independent only in amnestic MCI subjects and normal cognitive function subjects in T2DM. Adding the two groups to contrast analysis, the conclusion should be enriched and the results should be more convincing. Furthermore, the EEG signals were not analyzed when the patients were performing cognitive tasks. Recording data of more subjects, including normal elderly subjects, T2DM, and AD patients, and study of synchronization both at rest and performing cognitive tasks are still needed in further research.

## 5. Conclusion

In this paper, we analyzed the source EEG synchronization in amnesic MCI and normal cognitive function subjects in T2DM by three synchrony measures (*S*-estimator, SI, and GSI) and compared these values to explore the difference between the two groups. Results showed that cortical EEG source synchronization values illustrated significant difference between aMCI and control group. The aMCI group had significantly lower parietal and temporal *S*, SI, and GSI values in delta and beta2 bands. Occipital theta and beta2 synchronization values measured by the three methods were more significantly different between the two groups, especially in the beta2 band. Temporal and occipital theta *S* values were significantly positively correlated with Boston Name Testing. In sum, the synchronization of cortical EEG source was different between the amnestic and normal cognitive function subjects in T2DM and the synchronization was correlated with some cognitive functions.

## Figures and Tables

**Table 1:** Demographic characteristics and neuropsychological test scores and *P* values between aMCI and control groups in T2DM.

Factor	aMCI group	Control group	*P* value
Subjects	8	11	—
Gender (M/F)	5/3	5/6	—
Age (years)	70 ± 10.784	74.27 ± 3.349	NS
Education level (years)	13.88 ± 3.441	13.64 ± 2.541	NS
MMSE scores	26.88 ± 1.885	28.64 ± 0.674	0.034∗
MOCA scores	22 ± 1.852	27 ± 1.265	0.000∗∗
AVLT immediate recall	5.06 ± 1.664	7.78 ± 1.859	0.004∗∗
AVLT delayed recall	3.75 ± 3.105	8.82 ± 3.683	0.005∗∗
AVLT delay recognition	11 ± 3.117	13.91 ± 1.044	0.034∗
Boston Name Test	18.38 ± 1.188	19.82 ± 0.405	0.011∗
Semantic fluency	16.88 ± 3.314	18.18 ± 4.07	0.452
Trail Making test A	68.5 ± 19.878	60.64 ± 16.549	0.36
Trail Making test B	127.5 ± 62.551	102.91 ± 34.469	0.286
WAIS digit span test	11.5 ± 3.854	14.73 ± 2.24	0.034∗
IADL	1.13 ± 1.356	0.45 ± 1.508	0.333

Mean values ± standard errors of demographic characteristics and neuropsychological test scores in aMCI and control groups. *P* values were obtained comparing aMCI and control groups with independent samples *t*-test.

aMCI, amnestic mild cognitive impairment; AVLT, the Auditory Verbal Learning Test; F, female; IADL, Instrumental Activity of Daily Living Scale; M, male; MMSE, Mini-Mental State Examination; MOCA, Montreal Cognitive Assessment; NS, not significant; WAIS, Wechsler Adult Intelligence Scale; ∗indicates the *P* value < 0.05; ∗∗indicates the *P* value < 0.01.

**Table 2:** Brodmann areas into the regions of interest (ROIs).

ROI	Brodmann areas (left and right)
Frontal	8, 9, 10, 11, 44, 45, 46, 47
Central	1, 2, 3, 4, 6
Parietal	5, 7, 30, 39, 40, 43
Temporal	20, 21, 22, 37, 38, 41, 42
Occipital	17, 18, 19
